# Late presentation of incomplete jejunoileal junction atresia with recurrent midgut volvulus; A case report for chronic midgut volvulus in adults

**DOI:** 10.22088/cjim.14.2.380

**Published:** 2023

**Authors:** Abdolreza Emami, Yasser Asghari, Hasan Abedi Valukalaei, Mohammad Sakhaei

**Affiliations:** 1 Student Research Committee, School of Medicine, Babol University of Medical Sciences, Babol, Iran; 2 Cancer research center, Health research institute ,Department of Surgery, School of Medicine, Babol University of Medical Sciences, Babol, Iran; 3 Department of Internal Medicine, School of Medicine, Babol University of Medical Sciences, Babol, Iran; 4 Islamic Azad University Sari Branch, Sari Campus, Iran

**Keywords:** Midgut Malrotation, Volvulus, jejunal atresia, Adult, Congenital Abnormalities, Case Report

## Abstract

**Background:**

Despite the prevalence of intestinal malformation in Childs, late-onset during adulthood is rare and usually diagnosed incidentally. It may be present as subtle or vague abdominal pain following mid-gut volvulus. Computerized tomography may help diagnosis, but surgery remains the gold standard of diagnosis and treatment.

**Case Presentation:**

We presented a 24-year-old woman who complained of chronic intermittent abdominal pain, progressive food intolerance, and severe weight loss. Magnetic resonance enterography revealed dilated jejunum and collapsed ileum with slight bowel rotation around its mesentery (whirlpool sign), which was suggestive for mal-rotation of the intestine complicated by midgut volvulus, then the diagnosis was confirmed by laparotomy. During six months of follow-up after surgery, the patient's appetite improved significantly with eight kilograms of weight gain and resolution of abdominal pain.

**Conclusion:**

It may be a rationale to consider intestinal malformation as a differential diagnosis in a patient who complained of chronic abdominal pain with progressive weight loss, anorexia, and recurrent bowel obstructive symptoms.

Intestinal anomalies including malrotation with concomitant atresia is rare. Intestinal malrotation defines as the failure of the fetal intestine to complete rotation around the superior mesenteric axis (SMA) (1). Fifty-five percent of midgut malformation presents in the first week, and 80 percent shows in the first month of life (2). Intestinal atresia leads to complete obstruction of the bowel and could occur at any part of the gastrointestinal tract with the incident rate ranging from 1.3 to 3.5 per 10000 live births (3). The adult intestinal malformation is rare, with an estimated incidence rate of 0.2-0.5% (4), and in most cases is asymptomatic and usually diagnosed accidentally later in life (5). In symptomatic adults, it may presents acutely as bowel obstruction and intestinal ischemia associated with midgut volvulus or chronically as a cause of chronic intermittent obstruction with recurrent attacks or vague abdominal complaints extending back into childhood (6). Chronic symptoms are more subtle and non-specific and include intermittent abdominal pain, bloating, and food intolerance (7). Complex and varied clinical features can result in delayed diagnosis, and multiple refers to different clinical specialties at various times (8). The most common diagnostic imaging modality is computerized tomography (CT) scan with oral and intravenous contrast (8). Due to collapsed bowel loops surrounding the midgut mesenteric vasculature, the whirlpool sign is the classical CT scan associated with volvulus due to midgut malrotation(8). An upper gastrointestinal (UGI) series is the best examination to visualize the duodenum and, therefore, the gold standard for diagnosing intestinal malformation in adults (9).

Herein, we report a 24-year-old woman who complained of chronic abdominal pain, intermittent bloating, nausea and vomiting, progressive food intolerance, severe weight loss after her first vaginal delivery, and inconclusive referrals to different clinical specialties.

## Case Presentation

A 24-year-old household woman was admitted to our hospital with complaints of chronic abdominal pain, nausea and vomiting, and progressive food intolerance. The patient mentioned that her symptoms began since her childhood and was exacerbated after her first vaginal delivery five years ago. Her symptoms had gotten worse during the past three months. She described an intermittent colicky pain in the periumbilical area which got worse by eating and getting a little better with defecation. 

**Figure 1:**
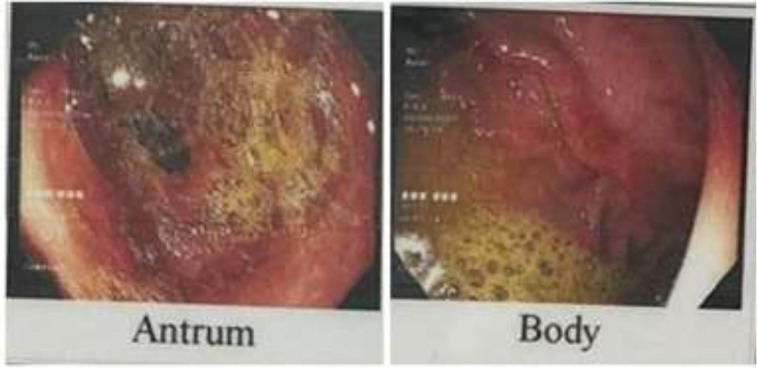
Upper gastroscopy revealed severe bile reflux gastritis, confirmed by histopathologic evaluation

She had intermittent nausea and vomiting especially post-prandial, containing food materials that got worse during the past three months. She mentioned progressive anorexia and food intolerance, which led to around 35 kilograms of weight loss during the past five years. She also mentioned an intermittent period of diarrhea and constipation in the past five years. During these years, she underwent upper gastroscopy, which revealed severe bile reflux gastritis (figure 1) with pathologic findings of moderate chronic gastritis and Helicobacter pylori colonization, and a colonoscopy revealed grossly normal mucosa up to the terminal ileum.

**Clinical finding: **Initial physical examinations were blood pressure of 120/80 mmHg without orthostatic hypotension, pulse rate of 95 beats per minute, respiratory rate of 20 per minute, and temperature of 36.8 ◦C. There was no abnormality in head, neck, and chest examination. Abdominal examination revealed upper abdominal distention with mild tenderness in the periumbilical and epigastric area without guarding or rebound tenderness. 

**Diagnostic assessment: **Initial Laboratory evaluations were normal except for mild anemia (hemoglobin of 11.7 mg/dl) and serum Na of 130 mmol/l (normal range of 136-146 mmol/l). Plasma levels of IgA, anti-Tissue transglutaminase Ab (IgG), anti-Tissue transglutaminase Ab (IgA), anti-Endomysial Ab (IgA), and anti-Endomysial Ab (IgG) were all in the normal range. So, no further evaluation was needed because of low clinical suspicion for celiac disease.

**Figure 2:**
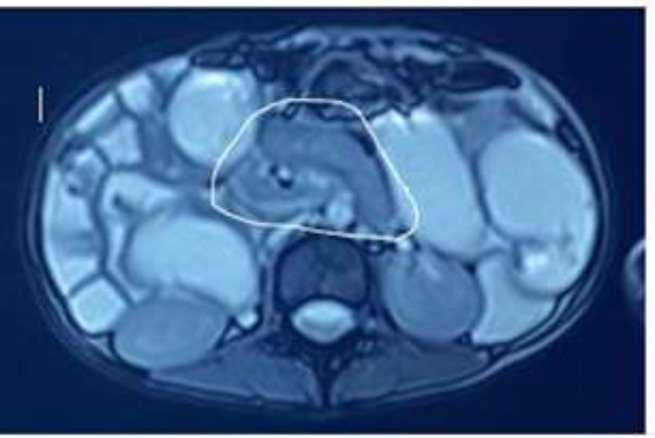
Magnetic Resonance Enterography revealed the dilated jejunum and proximal ileum with mid-small bowel rotating around its mesentery (whirlpool sign), (circular mark)

**Figure 3:**
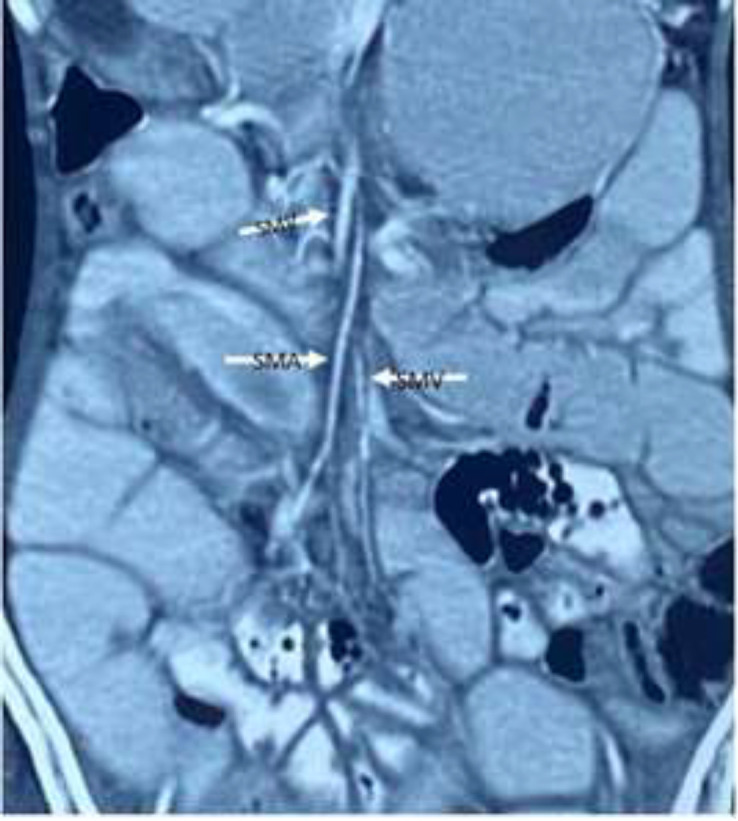
Magnetic Resonance Enterography revealed superior mesenteric vein rotating around the superior mesenteric artery (reverse relationship between the SMV and SMA), (arrow)

**Figure 4:**
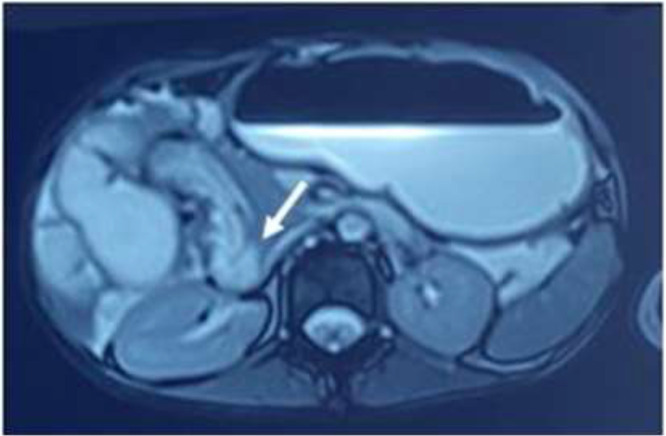
Magnetic Resonance Enterography revealed a failure of midline crossing of duodenal-jejunal junction (arrowhead) suggestive of mal-rotation complicated by midgut volvulus

Magnetic resonance enterography was requested and revealed dilated jejunum and collapsed ileum with mid-small bowel rotating around its mesentery (figure 2) and superior mesenteric vein turning around the superior mesenteric artery (figure 3) associated with failure to midline crossing of duodenal-jejunal junction (figure 4) suggestive of mal-rotation complicated by mid-gut volvulus. Due to the patient's situation, surgery consultation was requested, and based on the physical examination and previous assessment workups; a diagnostic laparoscopy was scheduled.

**Figure 5:**
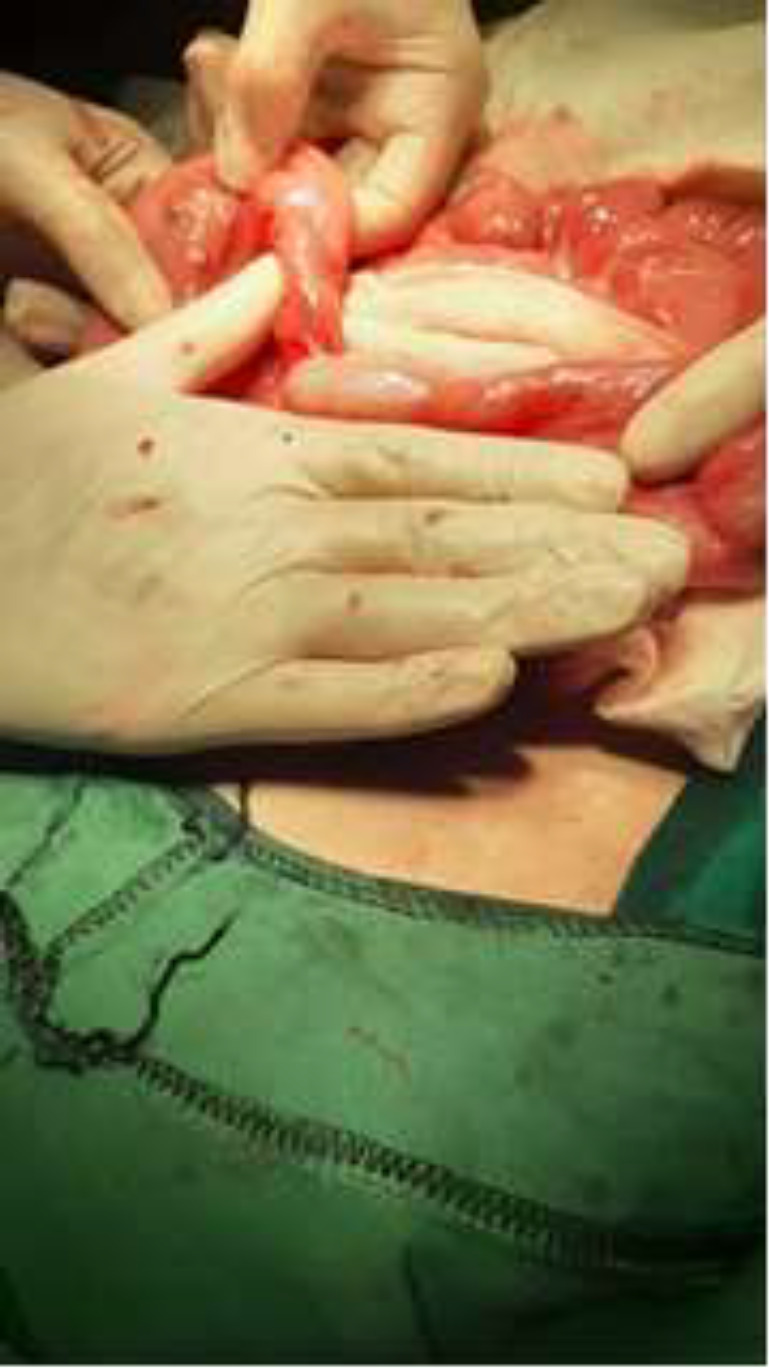
Site of stenosis and proximal dilation of the affected segment

**Therapeutic intervention: **During diagnostic laparoscopy, jejunal dilatation and ileal loop collapse, with segmental stenosis in the mid-portion of the small intestine distal to the jejunum was apparent, then the operation was converted to open laparotomy. There was 15-centimeter length spiral segment of the intestine with stenosis at the jejunoileal junction (apple peel sign) with volvulus of dilated, thickened wall, proximal segment around its mesentery and collapsed loop in the distal part suggestive for type 1 jejunoileal junction atresia accompanied by volvulus of proximal midgut (figure 5).

**Figure 6:**
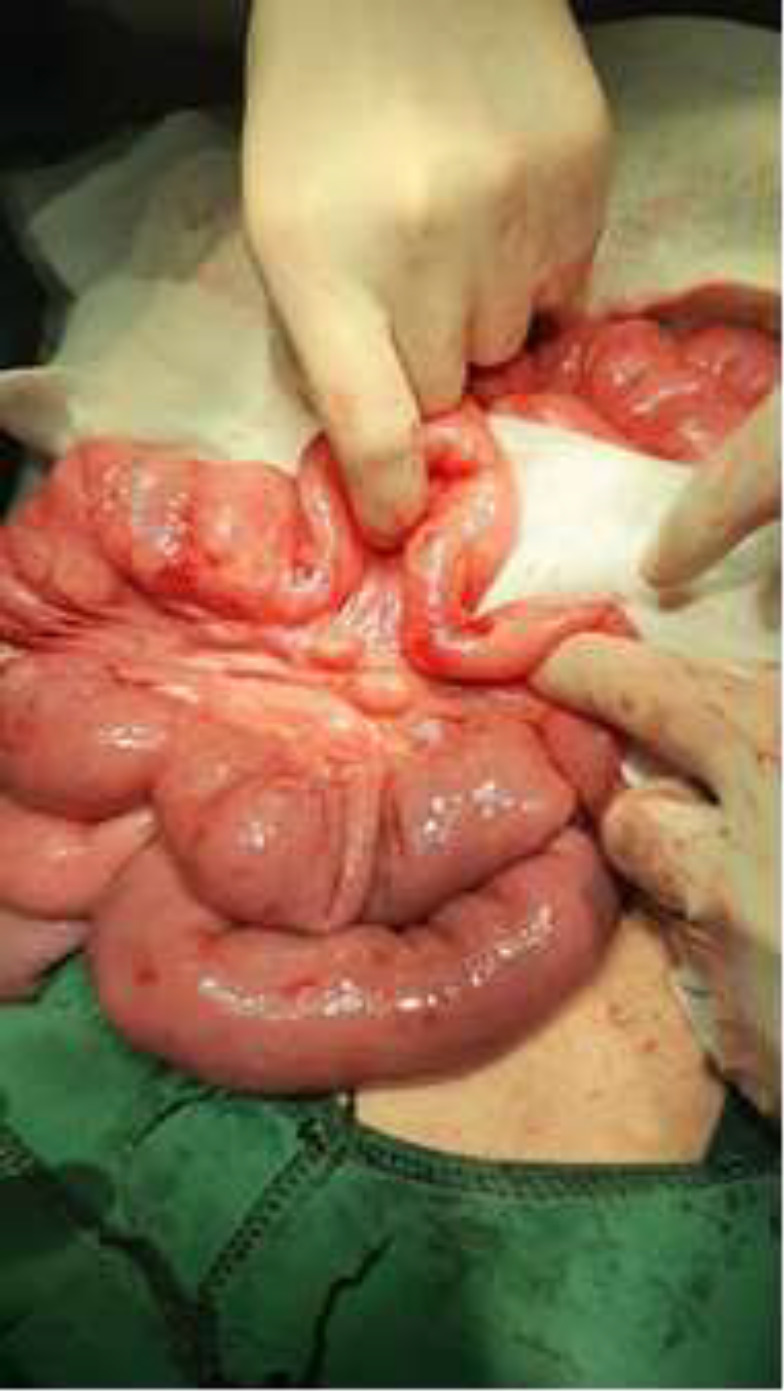
Prominent lymph nodes in the mesentery

**Figure 7:**
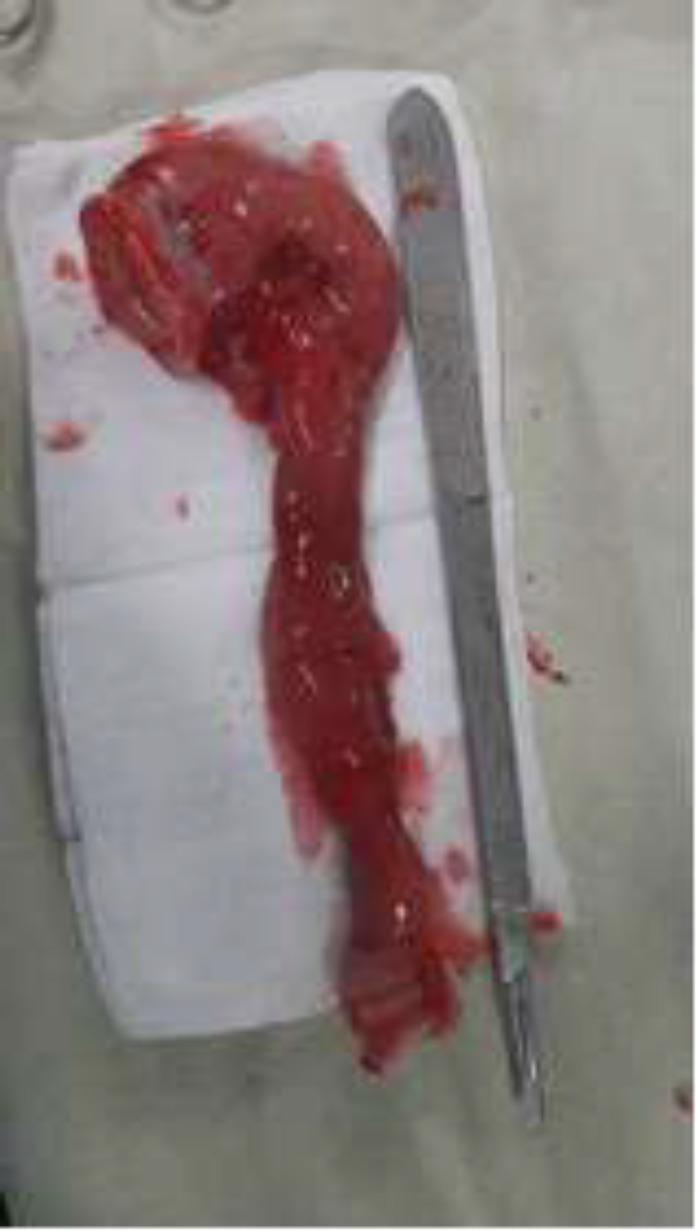
A resected segment of the stenotic intestine

 The mesentery of the affected segment was short and narrow, with enlarged lymph nodes and ecstatic lymphatic vessels (figure 6). Due to this finding, Devolvolation of the twisted bowel loop with resection of the stenotic part (figure 7) with its mesentery and enlarged lymph nodes following hand swan jejunoileal anastomosis in two layers was done. After surgery, the patient recovered with an excellent general condition and was discharged from the hospital ten days later.

**Follow-up and outcomes: **During six months of follow-up, the patient's appetite improved significantly with eight kilograms of weight gain, and abdominal pain resolved.

## Discussion

Incomplete intestine atresia accompanied with chronic midgut volvulus rarely presents in late adulthood (10-14). Patients often complain of intermittent chronic abdominal pain, change in bowel habits, and other non-specific symptoms (11). Our patient also presented with intermittent chronic abdominal pain, significant weight loss, and progressive anorexia. A spiral abdominal CT scan with contrast usually can show the abnormal anatomic location of the intestine and its relationship with surrounding tissue like the superior mesenteric vein and artery. Whirpool or whirl sign which describes the swirling appearance of bowel and mesentery twisted around the superior mesenteric arterial axis, is characteristic for intestinal malrotation(11). In our case, the presence of a whirlpool sign in the magnetic resonance enterography suggested the diagnosis, and diagnostic laparoscopy followed by laparotomy confirmed that. There are three forms of malrotation based on the Stringer classification; type 1 non-rotation, type 2 duodenal malrotation and type 3 duodenal plus caecal malrotation (13). There are four types of jejunoileal atresia; type 1 (mucosal web), type 2 (fibrous cord), type 3a (mesenteric gap defect), type 3b (apple peel), and type 4 (multiple atresias) (15). Surgery revealed type 1 malrotation plus type 1 jejunoileal junction atresia (false atresia; stenosis) in our case.

Despite the rare occurrence of midgut volvulus in adults as a late presentation of intestinal anomalies, it should be considered a differential diagnosis for chronic abdominal pain with non-specific symptoms. Appropriate diagnostic measures like abdominal CT scan with contrast or magnetic resonance enterography may be helpful to confirm the diagnosis. Still, surgery remains the gold standard for both diagnosis and treatment.
